# Activity of second-generation ALK inhibitors against crizotinib-resistant mutants in an NPM-ALK model compared to EML4-ALK

**DOI:** 10.1002/cam4.413

**Published:** 2015-02-26

**Authors:** Diletta Fontana, Monica Ceccon, Carlo Gambacorti-Passerini, Luca Mologni

**Affiliations:** 1 Department of Health Science, University of Milano-BicoccaMonza, Italy; 2 Section of Hematology, San Gerardo HospitalMonza, Italy

**Keywords:** Alectinib, ceritinib, crizotinib resistance, EML4-ALK, G1202R, NPM-ALK

## Abstract

Anaplastic lymphoma kinase (ALK) is a tyrosine kinase receptor involved in both solid and hematological tumors. About 80% of ALK-positive anaplastic large-cell lymphoma (ALCL) cases are characterized by the t(2;5)(p23;q35) translocation, encoding for the aberrant fusion protein nucleophosmin (NPM)-ALK, whereas 5% of non-small-cell lung cancer (NSCLC) patients carry the inv(2)(p21;p23) rearrangement, encoding for the echinoderm microtubule-associated protein-like 4 (EML4)-ALK fusion. The ALK/c-MET/ROS inhibitor crizotinib successfully improved the treatment of ALK-driven diseases. However, several cases of resistance appeared in NSCLC patients, and ALK amino acid substitutions were identified as a leading cause of resistance to crizotinib. Second-generation ALK inhibitors have been developed in order to overcome crizotinib resistance. In this work, we profiled in vitro the activity of crizotinib, AP26113, ASP3026, alectinib, and ceritinib against six mutated forms of ALK associated with clinical resistance to crizotinib (C1156Y, L1196M, L1152R, G1202R, G1269A, and S1206Y) and provide a classification of mutants according to their level of sensitivity/resistance to the drugs. Since the biological activity of ALK mutations extends beyond the specific type of fusion, both NPM-ALK- and EML4-ALK-positive cellular models were used. Our data revealed that most mutants may be targeted by using different inhibitors. One relevant exception is represented by the G1202R substitution, which was highly resistant to all drugs (>10-fold increased IC_50_ compared to wild type) and may represent the most challenging mutation to overcome. These results provide a prediction of cross-resistance of known crizotinib-resistant mutations against all second-generation tyrosine kinase inhibitors (TKIs) clinically available, and therefore could be a useful tool to help clinicians in the management of crizotinib-resistance cases.

## Introduction

Anaplastic lymphoma kinase (ALK) belongs to the insulin receptor protein-tyrosine kinase superfamily 1. ALK plays an important role in the nervous system development; indeed its expression is prominent in the brain and peripheral nervous system of developing embryos, but decreases rapidly after birth 2. The first evidence of ALK oncogenic properties emerged in 1994, when the fusion protein nucleophosmin (NPM)-ALK originated by the chromosomal translocation t(2;5)(p23;q35) was identified and associated with an aggressive form of non-Hodgkin T-cell lymphoma, known as anaplastic large-cell lymphoma (ALCL) 3. Several dysregulated or aberrant ALK forms have since been discovered as the cause of hematopoietic and non-hematopoietic malignancies, such as diffuse large B-cell lymphoma (DLBCL) 4, inflammatory myofibroblastic tumor (IMT) 5, neuroblastoma 6, anaplastic thyroid cancer 7, rhabdomyosarcoma 8, non-small-cell lung cancer (NSCLC) 9, and other diseases 10–17. In particular, about 5% of NSCLC cases carry the echinoderm microtubule-associated protein-like 4 (EML4)-ALK fusion protein resulting from inv(2)(p21;p23) 9.

Recently, the treatment of ALK-driven diseases was successfully improved by the development of crizotinib, an ALK/c-MET/ROS inhibitor approved in 2011 for the treatment of locally advanced or metastatic ALK-positive NSCLC 18, and currently in clinical trials for a variety of other ALK-related diseases including ALCL. Unfortunately, as expected from previous clinical experience with tyrosine kinase inhibitors (TKI), cases of resistance to crizotinib soon appeared in NSCLC patients. ALK point mutations, including C1156Y, L1196M 19, L1152R 20, G1269A 21, G1202R, and S1206Y amino acid substitutions and a 1151Tins insertion 22 were identified as the leading cause of crizotinib resistance. Additional mutations were found in specimens collected from patients affected by ALCL who developed resistance to crizotinib 23,24.

In order to overcome crizotinib resistance, second-generation small-molecule ALK inhibitors have been developed 25. AP26113, whose structure to date is unavailable, is currently undergoing phase I/II clinical trials, whereas ASP3026 is in phase I trials. Alectinib is in advanced phases of development and was recently approved in Japan for the treatment of ALK-positive NSCLC. Moreover, it has received breakthrough therapy designation by Food and Drug Administration (FDA) for patients with ALK-positive NSCLC who progressed on crizotinib. Ceritinib was approved in April 2014 for patients affected by metastatic ALK-positive NSCLC following treatment with crizotinib.

Although an increasing amount of data are available on clinical resistance to crizotinib and activity of new inhibitors in relapsed cases, a thorough direct comparison of the relative activity profiles of new drugs on crizotinib-resistant ALK mutants is still lacking. In this study, we focused on C1156Y, L1196M, L1152R, G1202R, G1269A, and S1206Y amino acid substitutions that have been identified in NSCLC patients progressing on crizotinib 19–22. Since EML4-ALK and NPM-ALK fusion proteins share the same functional ALK region, and given that the number of NSCLC patients treated with crizotinib is much higher than those affected by ALK-positive ALCL, it is possible that the same mutations conferring resistance to crizotinib identified in NSCLC patients, might also occur in ALCL cases and that the spectrum of mutations causing resistance to ALK inhibitors in NSCLC and lymphomas may at least in part overlap. Therefore, we decided to investigate the activity of all clinically relevant second-generation ALK inhibitors on a panel of 6 mutated forms of ALK, associated with crizotinib resistance in EML4-ALK-positive NSCLC patients, in an NPM-ALK-positive model. To further confirm the reliability of our model, we reproduced the same mutations in EML4-ALK, where they were originally detected. We used the Ba/F3 murine interleukin-3 (IL-3)-dependent pro-B-cell line to express either the wild type (WT) or the mutated forms of NPM-ALK and EML4-ALK, and we tested their sensitivity to crizotinib and all available second-generation ALK inhibitors.

Our study aims to profile the differences in the activity spectra of four ALK inhibitors against six crizotinib-resistant ALK mutations. Our data suggest that, for all tested mutants except G1202R, there is at least one valid therapeutic option, alternative to the standard second-line chemotherapy regimens. In fact, according to our data, G1202R may become a major obstacle to ALK inhibitor therapy, similar to the BCR-ABL T315I-mutant in Philadelphia-positive leukemia 26. With the purpose of pushing cancer treatment toward a more personalized therapy, the activity pattern presented in this study could help clinicians in the management of crizotinib-resistant cases in which relapse is due to the presence of known point mutation, and could drive medicinal chemistry efforts toward a focused research of new compounds able to overcome the most challenging resistance cases.

## Materials and Methods

### Chemicals and cell lines

Crizotinib (PF-2341066, Pfizer, New York, NY), AP26113 (Ariad Pharmaceuticals, Cambridge, MA), ASP3026 (Astellas Pharma, Tokyo, Japan), and ceritinib (LDK378, Novartis, Basel, Switzerland) were kindly provided by the companies. Alectinib (CH5424802, Chugai, Tokyo, Japan) was purchased by Selleck Chemicals (Houston, TX). All compounds were dissolved in dimethyl sulfoxide (DMSO; Sigma Chemical Co., St. Louis, MO) at 10 or 1 mmol/L concentration, according to solubility, aliquoted, and stored at −20°C.

Murine pro-B-cell line Ba/F3 was purchased from Leibniz-Institut DSMZ (Braunschweig, Germany), where they are routinely verified using polyphasic (genotypic and phenotypic) testing, and maintained in RPMI 1640 (Euroclone, Milano, Italy) supplemented with 9% Fetal Bovine Serum (Euroclone), 2 mmol/L l-glutamine (Euroclone), 100 U/mL penicillin (FBS, Euroclone), 100 *μ*g/mL streptomycin (Euroclone), 20 mmol/L 4-(2-hydroxyethyl)-1-piperazineethanesulfonic acid (HEPES, Euroclone), incubated in a humidified atmosphere at 37°C with 5% CO_2_. Ba/F3 cells’ medium was supplemented with Chinese hamster ovary cells’ supernatant (1:2000) as a source of IL-3. Cell number and viability were assessed by Trypan Blue (Sigma) count every 48 or 72 h.

### Site-directed mutagenesis, competent cells transformation, and Ba/F3 cell stable transfection

Ba/F3 EML4-ALK cells were obtained by transfecting Ba/F3 cell line with pCDH-CMV-MCS-EF1-Puro vector containing wild-type EML4-ALK (variant E6;A20) 21. This vector was kindly provided by Dr. R. C. Doebele (University of Colorado Anschutz Medical Campus, Aurora, CO). Ba/F3 EML4-ALK cell lines were maintained in the absence of IL-3, and kept under selection by adding 0.5 *μ*g/mL puromycin (Sigma), dissolved in water.

To establish Ba/F3 cells expressing mutated EML4-ALK, the pCDH-CMV-MCS-EF1-Puro vector-EML4-ALK plasmid was used as a template for in vitro site-directed mutagenesis. Plasmids coding for substitutions at positions C1156Y, L1196M, L1152R, G1202R, and S1206Y were generated. Site-directed mutagenesis was conducted using QuikChange II XL Site-Directed Mutagenesis Kit (Stratagene, La Jolla, CA) according to manufacturer’s instructions. For site-directed mutagenesis the following oligonucleotide sequences were used (mutated bases in bold):

C1156Y (FW): 5′-GAAGACGCTGCCTGAAGTGT**A**CTCTGAACAGGACGAACT-3′;
C1156Y (REV): 5′-AGTTCGTCCTGTTCAGAG**T**ACACTTCAGGCAGCGTCTTC-3′;
L1196M (FW): 5′-CTGCCCCGGTTCATCCTG**A**TGGAGCTCATGGCG-3′;
L1196M (REV):5′-CGCCATGAGCTCCA**T**CAGGATGAACCGGGGCAG-3′;
L1152R (FW): 5′-CTGTGAAGACGC**G**GCCTGAAGTGTGCTCTGAACAGG-3′;
L1152R (REV): 5′-CCTGTTCAGAGCACACTTCAGGC**C**GCGTCTTCACAG-3′;
G1202R (FW): 5′-GCTCATGGCGGGG**A**GAGACCTCAAGTCCTTC-3′;
G1202R (REV): 5′-GAAGGACTTGAGGTCTC**T**CCCCGCCATGAGC-3′;
S1206Y (FW): 5′-AGACCTCAAGT**A**CTTCCTCCGAGAGACCCGCC-3′;
S1206Y (REV): 5′-GGCGGGTCTCTCGGAGGAAG**T**ACTTGAGGTCT-3′.

Polymerase chain reaction (PCR) products were digested with DpnI (NEB, Ipswich, MA) for at least 1 h at 37°C, then 2 *μ*L of DpnI-treated PCR product were used for transformation of Stbl3 competent cells (Life Technologies, Carlsbad, CA) by heat shock according to manufacturer’s instructions. Transformed cells were plated on Luria-Bertani (LB)-ampicillin (50 *μ*g/mL) agar plates and incubated overnight at 37°C. Bacterial colonies picked from plates were grown overnight at 37° in LB with 50 *μ*g/mL ampicillin (Euroclone).

pCDH-CMV-MCS-EF1-Puro vector containing G1269A mutated EML4-ALK was provided by Dr. R. C. Doebele (University of Colorado).

Plasmids were recovered from 10 clones per cell line using Zyppy plasmid Miniprep Kit (Zymo Research, Irvine, CA), and mutations were confirmed by standard sequencing (Eurofins MWG Operon, Ebersberg, Germany), using the following primers:

C1156Y: 5′-CAGGAGCTGCAAGCCATGCAG-3′;
L1196M: 5′-CGACTACAACCCCAACTACTG-3′;
L1152R: 5′-CGACTACAACCCCAACTACTG-3′;
G1202R: 5′-GGAATGCCCAACGACCCAAG-3′;
G1269A: 5′-AGACCTCAAGTCCTTCCTCCG-3′;
S1206Y: 5′-GGAATGCCCAACGACCCAAG-3′.

Clones carrying the desired mutations were amplified and plasmids were extracted using NucleoBond Xtra Maxi EF (Macherey-Nagel, Bethlehem, PA).

Ten million Ba/F3 cells were electroporated with 30 *μ*g plasmid (270 V, 0.975 *μ*F) using a GenePulser XCell (Bio-Rad, Hercules, CA). Cells were initially maintained in complete medium supplemented by IL-3, and selected first with puromycin, followed by IL-3 withdrawal.

Ba/F3 NPM-ALK cells were obtained by Ba/F3 cell line electroporation as described earlier, with pcDNA3.0 vector containing wild-type NPM-ALK (the reference sequence for ALK sequence comparison used was NM_004304.4). This vector was kindly provided by Dr. S. W. Morris (St. Jude Children’s Research Hospital, Memphis, TN).

Ba/F3 NPM-ALK cell lines were maintained in the absence of IL-3, and grown under G418 selection (1 or 2 mg/mL, Euroclone). G418 was dissolved in DMSO (Sigma). The plasmid containing WT NPM-ALK was used as a template for in vitro site-directed mutagenesis, as described above.

Plasmids carrying the same point mutations described above were generated by using the same couples of primers, and amplified into TOP10 competent cells (Life Technologies).

In order to obtain G1269A mutation the following primers were used:

G1269A (FW): 5′-GAAGAGTGGCCAAGATTG**C**AGACTTCGGGATG-3′;
G1269A (REV): 5′-CATCCCGAAGTCT**G**CAATCTTGGCCACTCTTC-3′

Ba/F3 cells were transfected using the electroporation protocol described above and selected with G418, followed by IL-3 withdrawal.

### PCR

Five million cells were lysed in EuroGold TriFast (Euroclone) and total RNA was extracted according to manufacturer’s instructions. After DNase I treatment (Life Technologies), RNA was retrotranscribed using TaqMan Reverse Transcription reagents (Roche). cDNA obtained by retrotranscription was used to confirm the presence of mutations in each cell line. To this aim, standard PCR was conducted using FastStart High Fidelity PCR System (Roche), according to the manufacturer’s instructions.

EML4-ALK fragments were amplified using forward 5′-CACAACCTCTCCAAATACACA-3′ and reverse 5′-CAGAGATCTCTGTTCGAGTC-3′ primers.

NPM-ALK fragments were amplified using forward 5′-TGCATATTAGTGGACAGCAC-3′ and reverse 5′-CTGTAAACCAGGAGCCGTAC-3′ primers.

The purity of PCR fragments was checked by agarose gel electrophoresis. Single bands were purified on column using the QIAquick PCR Purification Kit (Qiagen, Hilden, Germany). Otherwise the fragment was isolated from gel using the QIAquick Gel Extraction Kit (Qiagen). Purified PCR products were sequenced by standard methods at Eurofins MWG Operon.

### Western blot and antibodies

One million cells were seeded in 12-well plate and drugs were added at indicated concentrations. After 4-h treatment, cells were harvested, washed once in phosphate buffered saline (PBS) at 4°C, and resuspended in Laemmli buffer supplemented with 10% β-mercaptoethanol (Sigma), 100 *μ*L/10^6^ cells. Lysates were denatured at 99°C for 20 min and then used for electrophoresis. Equal volumes (30 *μ*L) were loaded on 10% sodium dodecyl sulfate polyacrylamide gel electrophoresis (SDS-PAGE), transferred to nitrocellulose membrane Hybond ECL (GE Healthcare Life Science, Milano, Italy), and incubated overnight at 4°C with primary antibody (1:1000 dilution in bovine serum albumin [BSA] 2.5%, Roche). Secondary horseradish peroxidase-conjugated anti-mouse or anti-rabbit antibodies (1:2000) were incubated for 1 h at room temperature and then bands were visualized by chemiluminescence ECL (Thermo Scientific, Rockford, IL) as recommended by the manufacturer. Monoclonal anti-phospho-ALK (Y-1604) and monoclonal anti-ALK (31F12) antibodies were from Cell Signaling Technology (Danvers, MA), anti-ACTIN antibody was purchased from Sigma, anti-mouse and anti-rabbit antibodies were from Bio-Rad.

### Proliferation assay

Cells were seeded in 96-well plates (5000/well) and exposed to serial dilutions (1:3) of the indicated compounds, starting from 10 or 1 *μ*mol/L concentration. Vehicle alone was used as a control. After 72 h incubation at 37°C, methyl-^3^H-thymidine (Perkin Elmer, Waltham, MA) was added to the wells (1 *μ*Ci/well) and incubated for 8 h at 37°C. Cells were then harvested onto glass fiber filters (Perkin Elmer) using a Tomtec automated cell harvester and [methyl-^3^H]-thymidine incorporation was measured using a filter scintillation counter (1430 MicroBeta Wallac Trilux, Perkin Elmer). Each test was performed in triplicate and repeated at least twice.

### Software and data analysis

The detection of the desired point mutations in the established Ba/F3 cell lines and the sequence of primers used for mutagenesis and sequencing were obtained using Vector NTI (Life Technologies). Chromatograms were visualized using Chromas 2 (Technelysium, South Brisbane Queensland, Australia).

Western blot bands were visualized using Image Station 440 (Kodak, Rochester, NY), and protein expression levels were quantified using Carestream MI SE (Carestream Health, Inc., Rochester, NY).

Dose–response curves were analyzed using Graph-Pad Prism4 (GraphPad Software, Inc., La Jolla, CA). The IC_50_ value indicates the concentration of inhibitor that gives half maximal inhibition. Relative resistance (RR) index was calculated as the ratio between mutant and WT IC_50_ values. RR values were categorized in four resistance levels: sensitive (RR ≤ 2), moderately resistant (2 < RR ≤ 4), resistant (4 < RR ≤ 10), or highly resistant (RR > 10) 26.

## Results

### Establishment and characterization of Ba/F3 NPM-ALK cell lines

In order to obtain a reliable model to study the biological effects of clinically relevant ALK mutations, we expressed the WT and six mutated forms of human NPM-ALK in the pro-B murine Ba/F3 cell line. We obtained seven new cell lines: Ba/F3 NPM-ALK WT, and Ba/F3 NPM-ALK mutated cells carrying C1156Y, L1196M, L1152R, G1202R, G1269A, and S1206Y amino acid substitutions. For simplicity, we renamed the established Ba/F3 cell lines NA WT, NA C1156Y, NA L1196M, NA L1152R, NA G1202R, NA G1269A, and NA S1206Y, respectively. As expected, as a consequence of the oncogene expression, all of them were able to survive and proliferate in the absence of IL-3. In order to verify the presence of the desired mutation, the ALK region corresponding to the mutations was sequenced (Fig. S1A).

Resistance to crizotinib was verified by evaluating cell proliferation rates in the presence of increasing crizotinib doses for each cell line, using parental Ba/F3 cells as a negative control (Fig. S2). We defined for each mutant a RR index as the IC_50_ increase over NA WT, and classified mutants accordingly 26. The IC_50_ and RR values are shown in Table1. As expected, all mutated cell lines were resistant to crizotinib. Indeed, the observed IC_50_ value for NA WT cell lines was 42 nmol/L, while the corresponding values for mutated cells are in a range comprised between 121 and 902 nmol/L. All of the six mutants showed a RR value >2, confirming crizotinib resistance. As expected, parental Ba/F3 showed extremely high RR index (55.1), indicating that they are insensitive to the drug.

**Table 1:** IC_50_ values for crizotinib against parental Ba/F3, NPM-ALK WT, EML4-ALK WT, and mutated cells are reported

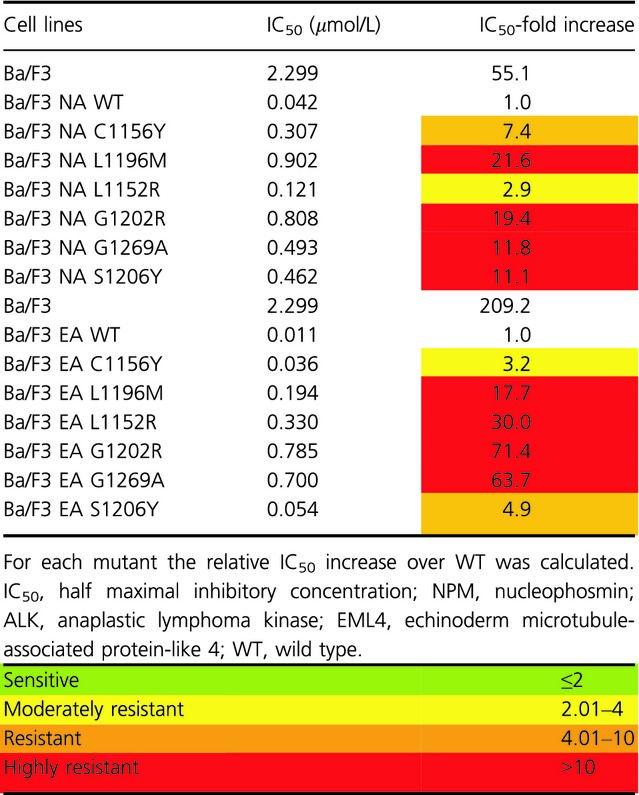

**Figure 1:**
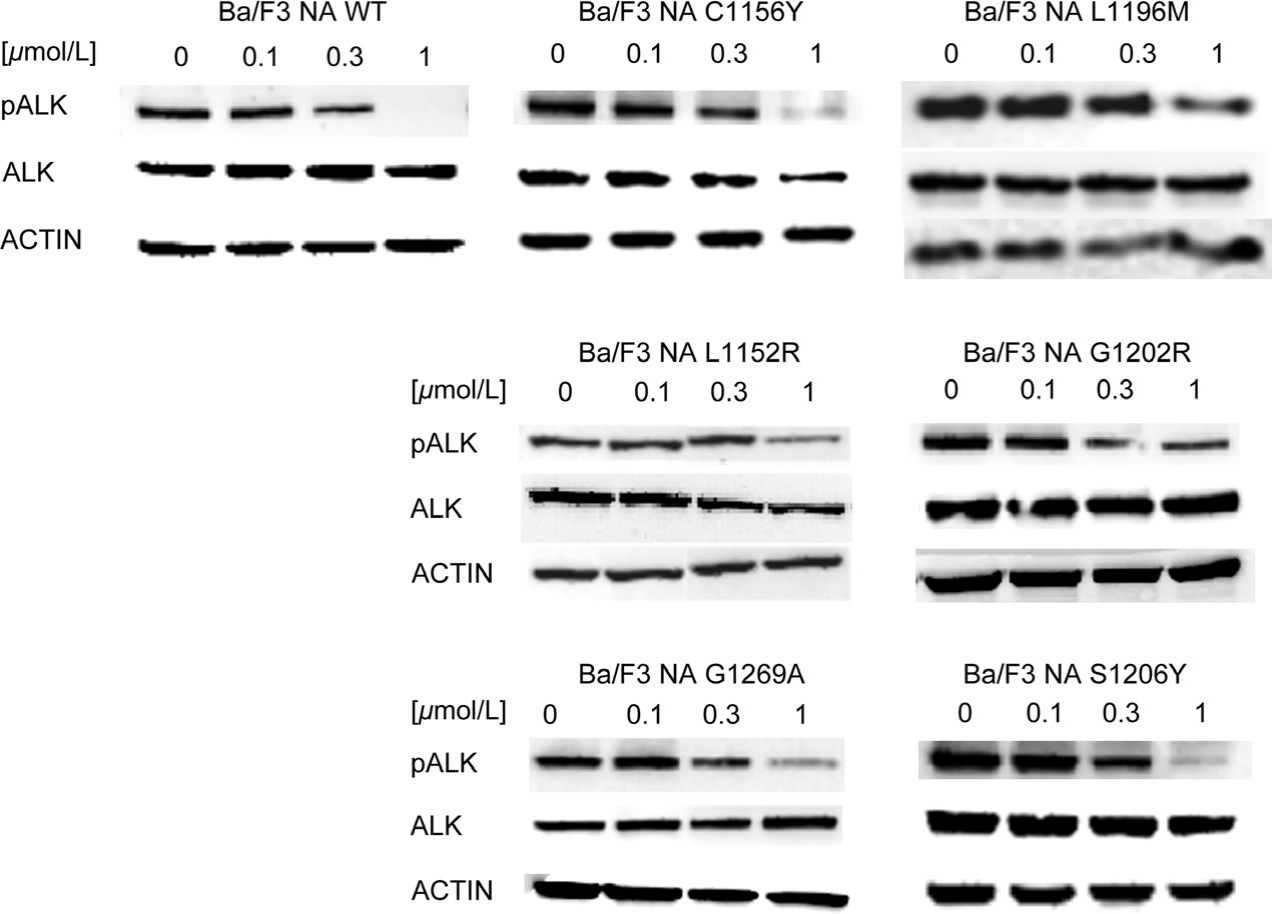
Resistance to crizotinib was investigated by western blot in Ba/F3 NPM-ALK cell lines. ALK phosphorylation status (Tyr1604) and total ALK expression levels were assessed after 4-h incubation with the indicated crizotinib concentrations. Actin was used as a loading control. ALK, anaplastic lymphoma kinase; NPM, nucleophosmin.

To confirm the data obtained with proliferation assays, we analyzed ALK phosphorylation status after treatment with increasing drug doses (Fig.1). Resistance to crizotinib was confirmed for all cell lines. Indeed, ALK phosphorylation in NA WT cells decreased starting from 0.1 *μ*mol/L, while it was comparable to the untreated sample in all mutated cell lines at this concentration. On the other hand, ALK phosphorylation was still present at 1 *μ*mol/L in NA L1196M, NA L1152R, NA G1202R, and NA G1269A mutants, while it was completely suppressed in NA WT cells.

### Cross-resistance of Ba/F3 NPM-ALK cell lines to other ALK inhibitors

The activity of AP26113, ASP3026, alectinib, and ceritinib against the six NA mutants was assessed by proliferation assay. Dose–response curves are shown in Figure S2. Again, the absolute IC_50_ and RR values were calculated and mutants classified in the same four categories (Tables2 and 3). With AP26113, four of six mutants (C1156Y, L1152R, G1269A, and S1206Y) were labeled as sensitive with RR values comprised between 0.4 and 1.9, whereas NA G1202R was highly resistant (RR = 13.3). The NA L1196M mutation was moderately resistant (RR value of 2.7). In contrast, only one of the mutants was fully sensitive to ASP3026 (NA S1206Y) while there were two resistant (L1196M and G1269A) and two highly resistant (L1152R and G1202R) mutants. NA C1156Y showed moderate resistance to ASP3026 (RR = 3.4). In the activity profile of alectinib, we found three sensitive mutations (C1156Y, L1196M, and S1206Y), and three with varying degrees of resistance (Tables3). In particular, G1202R was highly refractory to alectinib (RR = 27.9). Finally, two mutants were sensitive to ceritinib (G1269A and S1206Y), but the remaining four mutations were moderately resistant or highly resistant.

**Table 2:** IC_50_ values obtained by proliferation assay for AP26113, ASP3026, alectinib and ceritinib against parental, Ba/F3 NPM-ALK WT, EML4-ALK WT, and mutated cells are reported

Cell lines	IC_50_ (*μ*mol/L)			
	AP26113	ASP3026	Alectinib	Ceritinib
Ba/F3	>1	5.964	>10	>1
Ba/F3 NA WT	0.011	0.084	0.020	0.020
Ba/F3 NA C1156Y	0.016	0.284	0.038	0.071
Ba/F3 NA L1196M	0.030	0.682	0.032	0.042
Ba/F3 NA L1152R	0.004	2.763	0.047	0.288
Ba/F3 NA G1202R	0.149	1.177	0.549	0.277
Ba/F3 NA G1269A	0.011	0.364	0.104	0.019
Ba/F3 NA S1206Y	0.022	0.110	0.025	0.037
Ba/F3	>1	5.964	>10	>1
Ba/F3 EA WT	0.006	0.056	0.013	0.021
Ba/F3 EA C1156Y	0.005	0.090	0.010	0.026
Ba/F3 EA L1196M	0.008	0.141	0.022	0.019
Ba/F3 EA L1152R	0.001	0.514	0.017	0.099
Ba/F3 EA G1202R	0.325	1.210	0.868	0.467
Ba/F3 EA G1269A	0.009	0.118	0.084	0.033
Ba/F3 EA S1206Y	0.021	0.118	0.037	0.038

1 IC_50_, half maximal inhibitory concentration; NPM, nucleophosmin; ALK, anaplastic lymphoma kinase; EML4, echinoderm microtubule-associated protein-like 4; WT, wild type.

**Table 3:** For each mutant the relative IC_50_ increases over WT was calculated

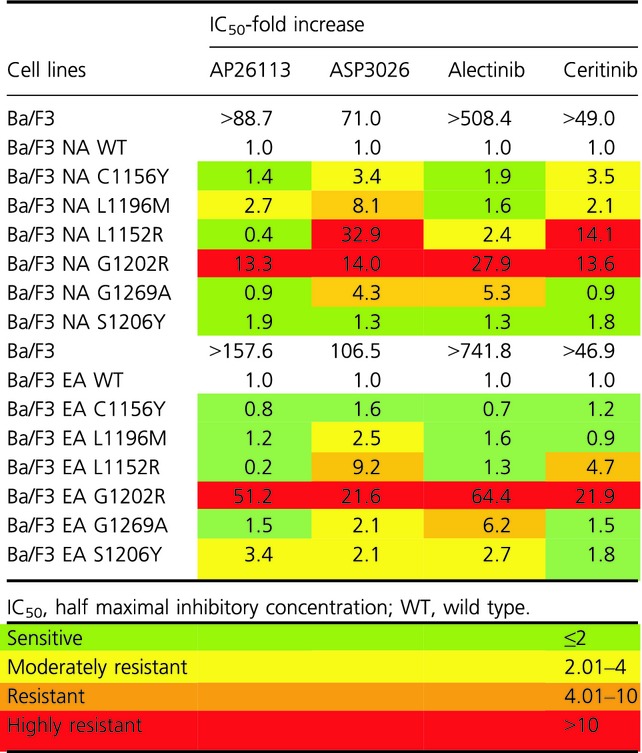

Interestingly, none of the considered second-generation drugs was able to inhibit the NA G1202R mutant that scored as highly resistant to all compounds. On the other hand, all second-generation inhibitors were able to target the S1206Y substitution.

**Figure 2:**
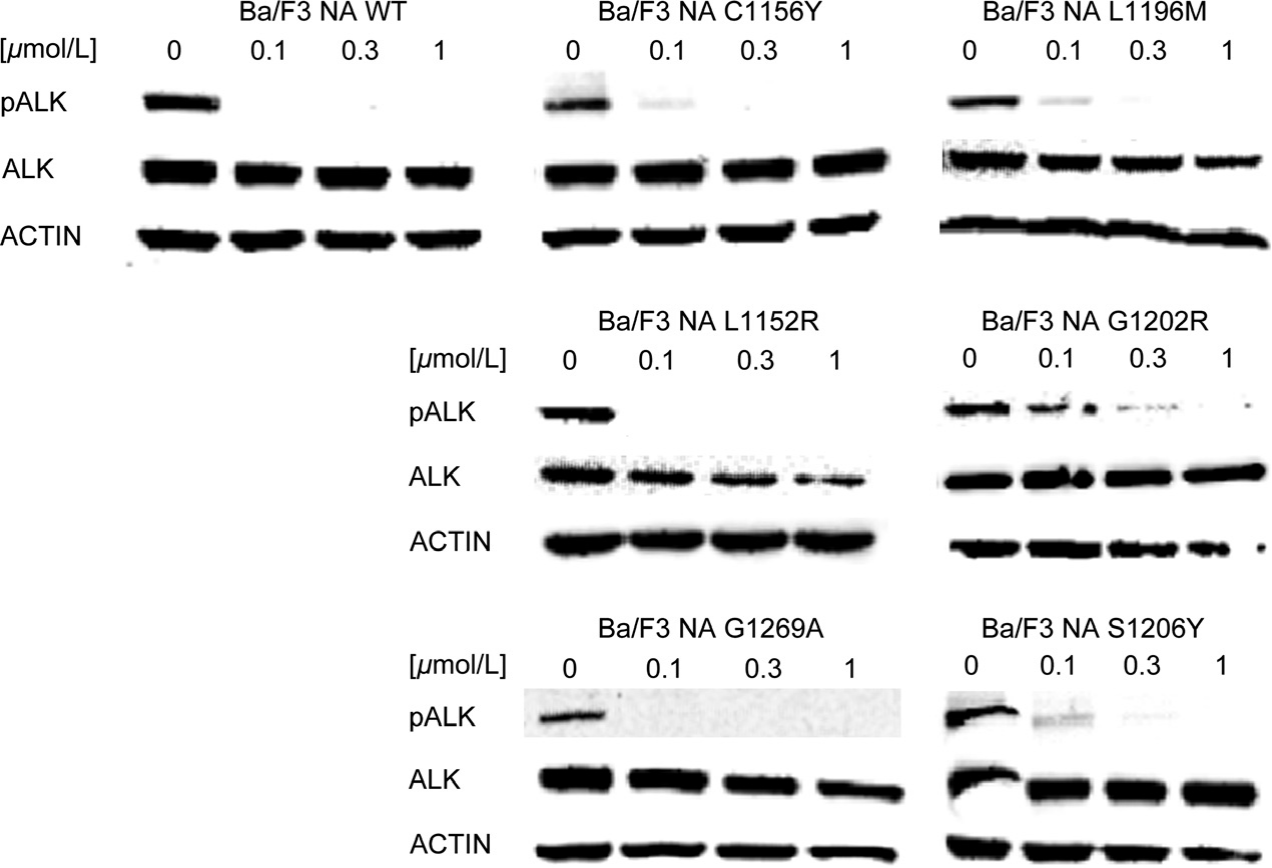
AP26113 activity was investigated by western blot in Ba/F3 NPM-ALK cell lines. ALK phosphorylation status (Tyr1604), total ALK, and the loading control actin were assessed by western blot analysis after 4-h incubation with the indicated concentrations of the drug. ALK, anaplastic lymphoma kinase; NPM, nucleophosmin.

**Figure 3:**
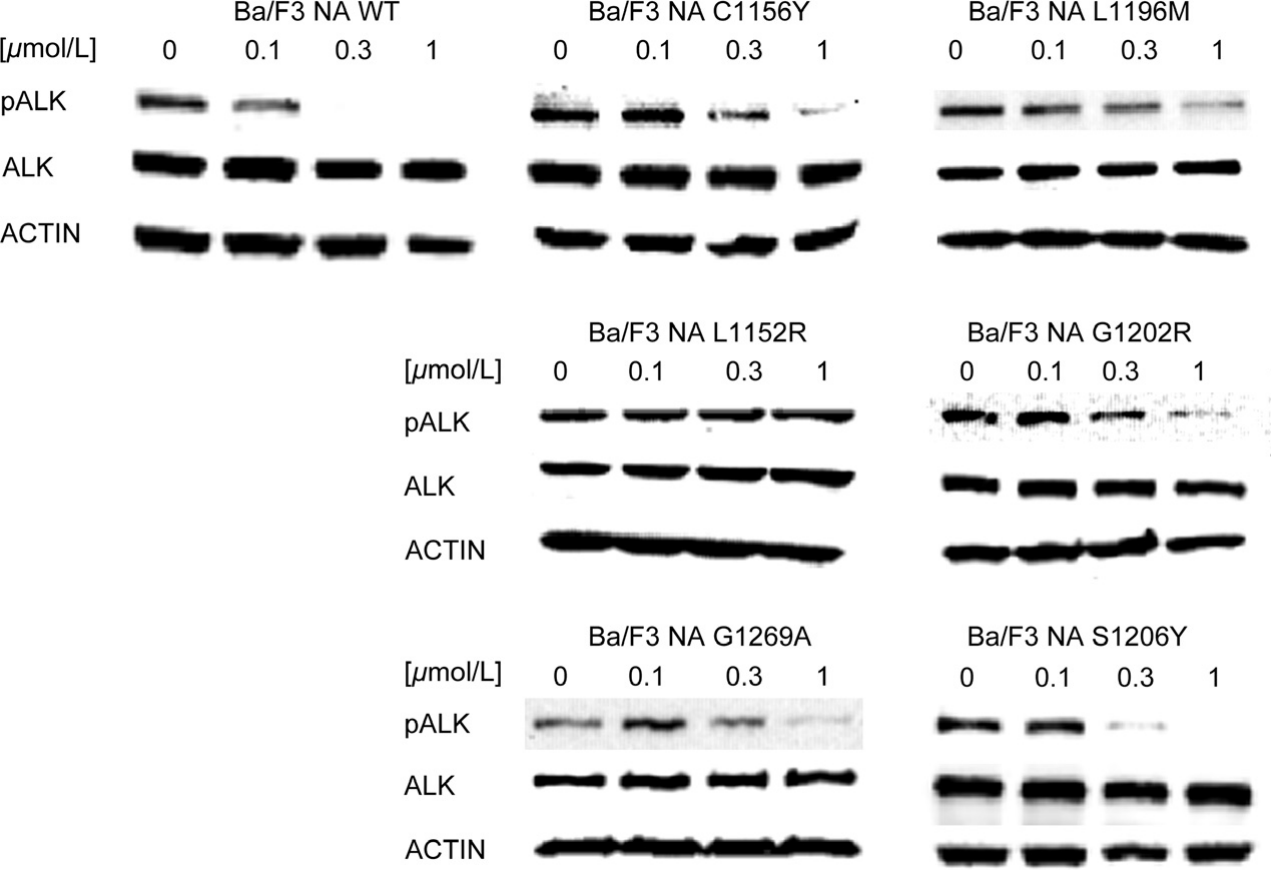
ASP3026 activity was investigated by western blot in Ba/F3 NPM-ALK cell lines. ALK phosphorylation status (Tyr1604), total ALK, and the loading control actin were assessed by western blot analysis after 4-h incubation with the indicated concentrations of the drug. ALK, anaplastic lymphoma kinase; NPM, nucleophosmin.

To confirm these data, we checked ALK phosphorylation status in all cell lines against all the drugs described (Figs.5). Upon treatment with AP26113 (Fig.2), ALK phosphorylation is completely abrogated or strongly inhibited at 0.1 *μ*mol/L, except for NA G1202R. ALK phosphorylation data obtained for this mutant are consistent with the IC_50_ values obtained by proliferation assay (0.149 *μ*mol/L), as weak phosphorylation signal is still observed at 0.3 *μ*mol/L while it is completely abrogated at 1 *μ*mol/L. In the presence of ASP3026 (Fig.3), phosphorylated ALK was still present at 1 *μ*mol/L in all mutants except S1206Y, thus confirming proliferation assay data. NA S1206Y phospho-ALK pattern is similar to the WT, indeed this mutant was sensitive to ASP3026.

**Figure 4:**
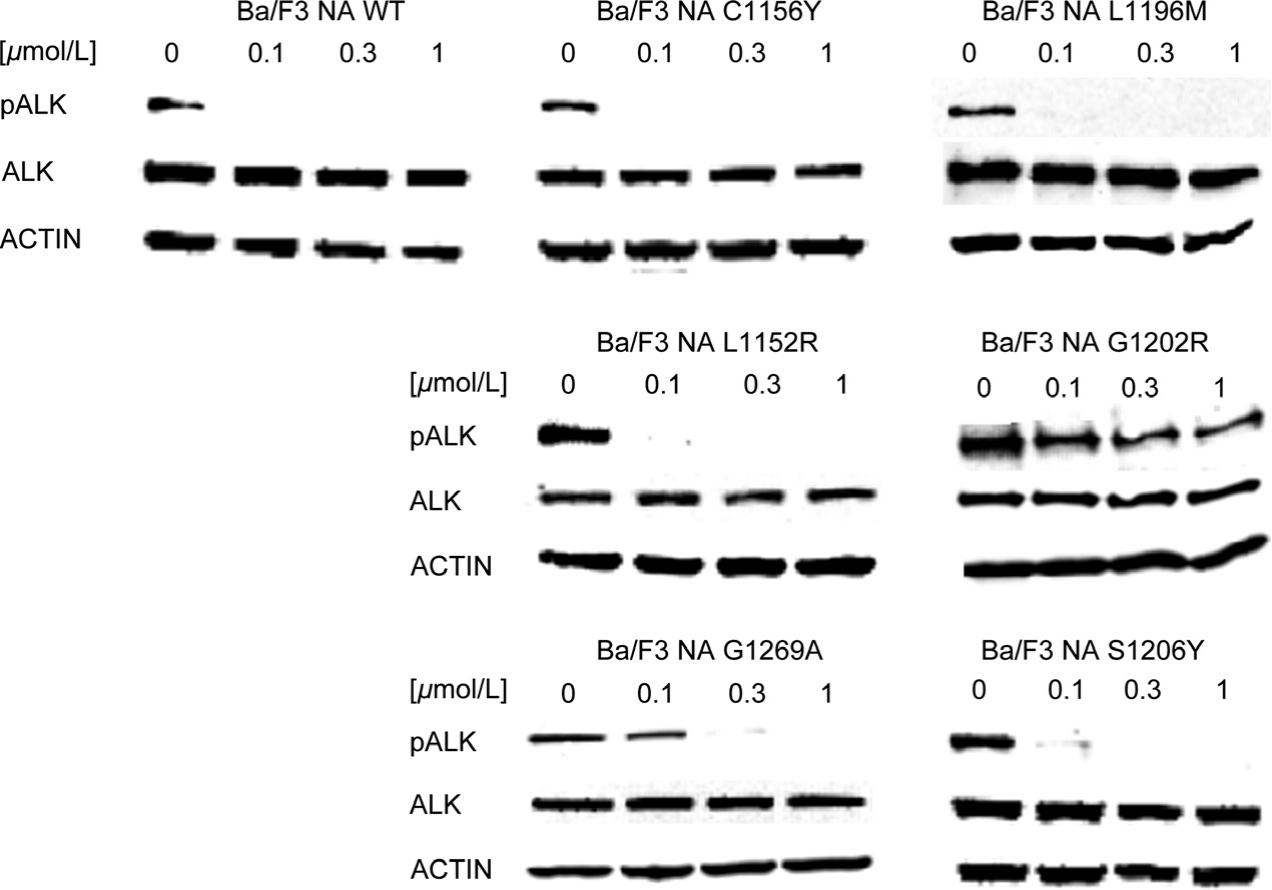
Alectinib activity was investigated by western blot in Ba/F3 NPM-ALK cell lines. ALK phosphorylation status (Tyr1604), total ALK, and the loading control actin were assessed by western blot analysis after 4-h incubation with the indicated concentrations of the drug. ALK, anaplastic lymphoma kinase; NPM, nucleophosmin.

**Figure 5:**
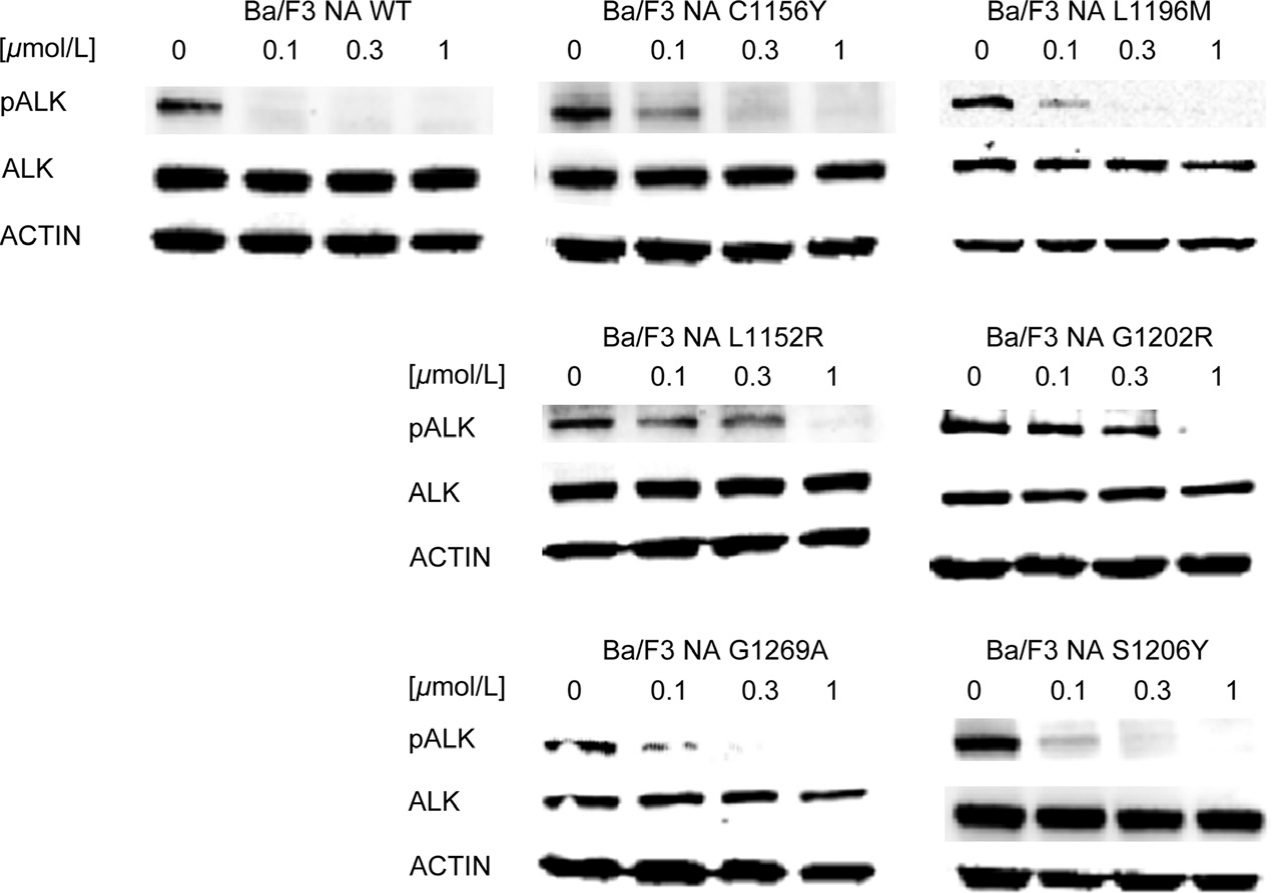
Ceritinib activity was investigated by western blot in Ba/F3 NPM-ALK cell lines. ALK phosphorylation status (Tyr1604), total ALK, and the loading control actin were assessed by western blot analysis after 4-h incubation with the indicated concentrations of the drug. ALK, anaplastic lymphoma kinase; NPM, nucleophosmin.

Analysis of ALK phosphorylation signal after alectinib administration (Fig.4) revealed a stark decrease in phosphorylation already at 0.1 *μ*mol/L, except for NA G1202R and NA G1269A mutants. The former resulted highly resistant to this drug: indeed ALK phosphorylation is still present at 1 *μ*mol/L; the latter showed no ALK phosphorylation starting from 0.3 *μ*mol/L, confirming proliferation data. Western blot also confirmed cell growth results for ceritinib (Fig.5). Indeed, whereas in NA WT and sensitive mutants phosphorylated ALK already decreases at 0.1 *μ*mol/L, persistent ALK phosphorylation is observed at 0.3 *μ*mol/L in NA L1152R and NA G1202R mutants. Western data were consistent with calculated RR indexes also for the remaining mutants.

### Establishment and characterization of Ba/F3 EML4-ALK cell lines

Thus far, drug resistance was profiled in the context of NPM-ALK fusion. Although the catalytic domain of ALK should be an independent structural and functional unit, we cannot rule out fusion type-dependent variations in drug sensitivity. In order to recapitulate the biological role of the original crizotinib-resistant mutations, we introduced them in the fusion protein where they were detected for the first time. The WT and mutated EML4-ALK forms were introduced in Ba/F3 cells, to obtain seven new cell lines (referred to as EA WT, EA C1156Y, EA L1196M, EA L1152R, EA G1202R, EA G1269A, and EA S1206Y).

**Figure 6:**
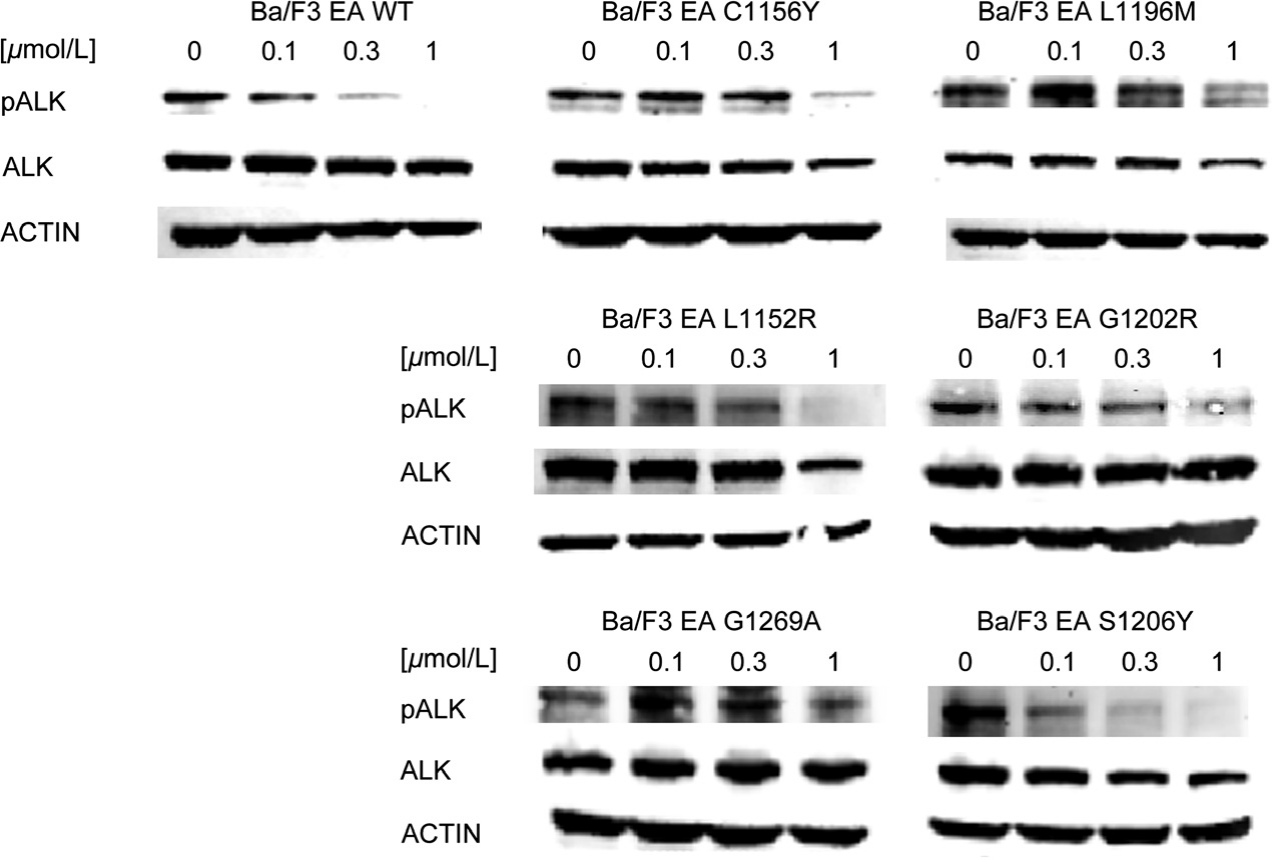
Resistance to crizotinib was investigated by western blot in Ba/F3 EML4-ALK cell lines. ALK phosphorylation status (Tyr1604) and total ALK expression levels were assessed after 4-h incubation with the indicated crizotinib concentrations. Actin was used as a loading control. ALK, anaplastic lymphoma kinase; NPM, nucleophosmin; EML4, echinoderm microtubule-associated protein-like 4.

After verifying the presence of the desired mutation (Fig. S1B), crizotinib resistance was evaluated (Fig. S2, Table1). As expected, all mutated cell lines were resistant to crizotinib. The IC_50_ value for EA WT was 11 nmol/L, while the corresponding values for mutated cells lie in a range comprised between 36 and 785 nmol/L. Among the six mutations considered, all showed a RR values >2, thus confirming crizotinib resistance. To further confirm the data, ALK phosphorylation was studied after treatment with increasing crizotinib concentrations (Fig.6). Phospho-ALK signal in EA WT cells decreased starting from 0.1 *μ*mol/L and significantly from 0.3 *μ*mol/L, while it was comparable to the untreated sample in EA C1156Y, EA L1196M, EA L1152R, EA G1202R, and EA G1269A cell lines at the same dose. Surprisingly, ALK phosphorylation inhibition in EA S1206Y mutant was similar to WT. Notably, this mutant showed limited resistance in cell proliferation.

### Cross-resistance of Ba/F3 EML4-ALK cell lines to other ALK inhibitors

Having established that the Ba/F3 EML4-ALK cell lines represent a reliable model, we sought to determine their sensitivity to the second-generation ALK inhibitors by proliferation assays (Fig. S2). The IC_50_ results are reported in Table2, whereas RR index values are listed in Table3. In the activity profile of AP26113, EA C1156Y, EA L1196M, EA L1152R, and EA G1269A mutants were classified as sensitive, whereas EA G1202R was again highly resistant (RR index of 51.2). The EA S1206Y mutation was classified as moderately resistant. Regarding ASP3026, only EA C1156Y mutant was sensitive, whereas the others were resistant. Three sensitive (EA C1156Y, EA L1196M, and EA L1152R) and three resistant (EA G1202R, EA G1269A, and EA S1206Y) mutations were observed with alectinib, while ceritinib targeted four of six mutants (EA L1152R was resistant, whereas EA G1202R was highly resistant with RR value of 21.9).

These data indicate that, even within the EML4-ALK fusion, the G1202R substitution could not be inhibited by any of the considered drugs. In contrast, all new ALK inhibitors were able to target the C1156Y amino acid change at very low doses (ranging from 5 nmol/L for AP26113 to 90 nmol/L for ASP3026), well-tolerated in patients 27.

## Discussion

In the last few years, the treatment of ALK-driven diseases changed significantly due to the development of crizotinib. However, most patients acquired drug resistance and relapsed. Therefore, second-generation ALK inhibitors have been developed in order to overcome crizotinib resistance. Most information currently available on the clinical efficacy and causes of relapse on crizotinib treatment are collected in the context of EML4-ALK-positive non-small-cell lung cancer, which is the most common ALK-related disease, whereas little is known about other ALK-positive malignancies.

We focused our attention on the oncogenic fusion protein NPM-ALK, originating from the reciprocal (2;5) translocation, that is responsible for about 70–80% of ALK-positive anaplastic large-cell lymphoma cases. Our study investigated, in an NPM-ALK-positive cellular model, the differences in activity within a panel of clinically relevant second-generation ALK inhibitors (AP26113, ASP3026, alectinib, and ceritinib) against six mutated forms of ALK (NA C1156Y, NA L1196M, NA L1152R, NA G1202R, NA G1269A, NA S1206Y) associated with crizotinib resistance in ALK-positive NSCLC patients. We took advantage of the broadly used murine pro-B-cell line Ba/F3. This simple, but efficient, model allowed us to study the actual biological role of each single mutation, since they are pulled out from the context where they originated.

Resistance to crizotinib was confirmed in our Ba/F3 NPM-ALK model by proliferation assay and by ALK tyrosine 1604 phosphorylation status (Table1, Figs.1 and S2). Some discrepancies between the two assays warrant a comment: for example, ALK phosphorylation was still present at 1 *μ*mol/L in NA L1152R mutant, and this does not fully correlate with cell growth data, which indicated only moderate resistance to crizotinib (IC_50_ = 121 nmol/L). However, western blot is known to be a semiquantitative method and therefore unsuitable for precise quantification. Overall, this mutation can be assigned a moderate level of resistance, which can be anyway sufficient to cause relapse in the clinical setting.

We then profiled the activity of four second-generation ALK inhibitors against six clinical ALK mutants, in both NPM-ALK and EML4-ALK fusions (Fig. S2). Our data highlight substantial differences among the considered drugs: cross-resistance experiments revealed that, except for G1202R, all point mutations may be targeted simply by changing inhibitors, already available in the clinic. All IC_50_ values calculated by proliferation test were confirmed by western blot to verify that resistance was really due to a specific impairment in ALK targeting. Proliferation assay sensitivity is much higher than western blot; however, we could in general establish a direct correlation between IC_50_ values and ALK phosphorylation status. Crizotinib resistance was less evident in the case of EA S1206Y where ALK phosphorylation began to decrease at 0.1 *μ*mol/L (Fig.6), but this is consistent with the IC_50_ value (Table1) and with data already published by others 22, and nevertheless S1206Y was able to confer clinical as well as in vitro crizotinib resistance. Notably, the resistance profile we obtained for ceritinib and alectinib is consistent with data recently published 28–30. The NA G1202R mutant was highly resistant to all new inhibitors tested, even though AP26113 (Fig.2) and ceritinib (Fig.5) at 1 *μ*mol/L suppressed ALK phosphorylation signal. These results are consistent with the resistance profile obtained by proliferation assays (Tables2 and 2) and are not in contrast with classification of this mutant as “highly resistant,” if we consider this definition as a RR index to be compared with WT. Moreover, such a concentration (1 *μ*mol/L) lies at the upper limit of plasma levels for these drugs in patients, suggesting that it may not be achieved in a majority of individuals 27.

The comparison between NPM-ALK and the EML4-ALK counterpart, where all mutations analyzed were found, allowed us to determine if ALK fusion partner has a biological role in drug resistance. By comparing the activity profile of NPM-ALK and EML4-ALK, we concluded that ALK fusion partner does not greatly affect drug responses, consistent with the fact that all considered mutations are localized inside the ALK kinase domain that is shared by both fusion proteins. However, Heuckmann et al. reported a different drugs sensitivity of various EML4-ALK variants 31, even though this phenomenon has not been observed in clinical setting. In our experiments, EA L1152R, EA G1202R, and EA G1269A showed higher RR to crizotinib than the NPM-ALK counterpart. Notably a difference in fusion protein expression level is appreciable between EA and NA L1152R and G1269A mutants, which may account for RR variation. Moreover, the RR index difference observed (Table1) may in part be due to the IC_50_ values obtained for the WT (11 vs. 42 nmol/L), used for RR normalization. These values inevitably weigh on the final RR index, but we do not consider this difference significant from a biological point of view. Indeed, upon treatment with 1 *μ*mol/L crizotinib, the level of residual ALK phosphorylation in NPM-ALK mutants (Fig.1) appeared even higher than in the corresponding EML4-ALK cells (Fig.6). Likely, these differences in ALK phosphorylation can be attributed to the semiquantitative character of western blot technique. Overall, by combining all the data, we infer that the resistance profile is not grossly altered by the different fusion partner, although we cannot conclusively rule out the possibility that some of the observed differences between the NPM-ALK model and its EML4-ALK counterpart are due to the peculiar fusion protein analyzed.

The gatekeeper NA L1196M has been soon recognized as highly resistant to crizotinib 19. From a molecular point of view, ALK L1196M corresponds to the highly TKI resistant BCR-ABL T315I 32, c-KIT T670I 33, and EGFR T790M 34,35 mutations. Therefore, it was believed to become the most difficult ALK mutant and much effort has been put to overcome its effects. In fact, in our cells, both NA L1196M and EA L1196M could be targeted by one or more new ALK inhibitors. NA L1196M could be treated administrating alectinib, and probably also ceritinib, which showed a limited resistance (RR = 2.1), still active at a low nanomolar dose. For EA L1196M, there are even three new drugs (alectinib and ceritinib, but also AP26113) able to overcome crizotinib resistance.

While the gatekeeper mutant may not be a major issue with the new inhibitors, the G1202R substitution seems to be highly resistant not only to crizotinib but also to all second-generation inhibitors, suggesting that this may represent the most challenging mutation to target. These results are consistent with published data, in which EA G1202R resulted highly resistant to the tested inhibitors, both in vitro and in vivo. Indeed, G1202R exhibited a high level of resistance to both ASP3026 and alectinib in a Ba/F3-EML4-ALK model 22,28. Moreover, this mutant conferred resistance to ceritinib both in vitro (Ba/F3 cells) and in vivo (H2228 crizotinib-resistant xenograft carrying the G1202R mutation) 29. Furthermore, G1202R was found in two patients’ biopsies who relapsed after ceritinib administration and in these cancers this mutant was selected by ceritinib 29. Again, the same mutation was identified in a patient who progressed on crizotinib treatment and continued disease progression upon alectinib administration 36. While these anecdotal clinical cases suggest that the G1202R mutant may be refractory to multiple therapies, our work provides a systematic analysis of the relative potency of all drugs against this mutation. G1202R mutation is located into ALK active site, facing the drug binding pocket, and corresponds to the BCR-ABL G321W 37 that was predicted to confer imatinib resistance in a random mutagenesis screening but never found in patients, and also corresponds to the ROS1 G2032R mutation that was identified in a crizotinib-resistant CD74-ROS1 metastatic lung adenocarcinoma 38. Substitution with an arginine causes a drug rearrangement responsible for the stabilization of the 1259-1264 loop and a consequent loss of conformational entropy 39, thus favoring an incorrect crizotinib orientation and reducing drug affinity. Whether this shift in drug positioning inside the pocket is also responsible for the observed resistance of G1202R to the other compounds is unknown.

On the other hand, all other mutants analyzed, for which no activating mutations at these positions in other oncogenic tyrosine kinases have been reported, were sensitive to at least one second-generation TKI. C1156Y is located nearby the alpha-C helix (1158-1173). Despite residue 1156 is far from crizotinib binding site, this substitution is sufficient to induce a conformational change in the binding cavity that globally weakens van der Waal interactions between the drug and the protein, thus weakening crizotinib binding activity 40. Consistent with our data, substitution with a phenylalanine, an amino acid that is structurally close to tyrosine, was identified in a NPM-ALK-positive in vitro model of ASP3026 resistance (Mologni Luca, unpubl. ms.). In an EML4-ALK context, C1156Y was sensitive to all drugs, while in NPM-ALK it was slightly resistant to ASP3026 and ceritinib. Even though the ALK fragment is maintained in both fusions, we cannot exclude that the difference in drug sensitivity is due to the different partner. The L1152R change causes a repulsion and hence moving out of K1150, which in turn blocks crizotinib binding 39. Recently, a proline substitution in the same residue has been shown to be responsible for ceritinib resistance 29. This can be consistent with the resistance profile we found against the same drug in both lymphoma and lung cancer context. Residue S1206 lies close to the crizotinib binding site and again its substitution with a tyrosine causes an incorrect drug location that is pulled more outside the pocket 39. Substitution with a cysteine was found to confer AP26113 resistance in a NPM-ALK-positive in vitro model 41. Finally, the glycine positioned at 1269 is located into the ATP binding pocket, therefore its replacement with a bulkier amino acid such as an alanine could be sufficient to disrupt the drug–protein interaction 21.

Recently a novel ALK F1174V mutation has been found in an ALK-positive NSCLC patient who, after a first partial response, progressed on crizotinib 36. We found the same substitution, in combination with L1198F, in an NPM-ALK-positive in vitro model of AP26113 resistance 41.

Direct comparison between the activity of structurally unrelated inhibitors against all recurrent crizotinib-resistant mutations is an important knowledge for clinical practice, since it is a useful tool to direct the oncologist in the management of ALK-related malignancies. Indeed the clinical availability of several compounds can be an important plan of action to hit a tumor that is known to be intrinsically heterogeneous.

Collectively, our results provide a reliable prediction about the cross-resistance of known crizotinib-resistant mutations across all second-generation TKIs clinically available, with the purpose to direct clinicians toward a more personalized therapy, thus enhancing the probability of success.

## Supporting Information

**Figure S1.** Chromatograms related to all mutations inserted in Ba/F3 cell line (A) NA and (B) EA are shown and compared to the WT. Asterisks highlight the mutated base.

**Figure S2.** Dose–response curves obtained by proliferation assay after 72-h treatment with serial drugs dilutions. The graphs are representative of three or more experiments.

## References

[b1] Lemmon MA, Schlessinger J (2010). Cell signaling by receptor tyrosine kinases. Cell.

[b2] Iwahara T, Fujimoto J, Wen DZ, Cupples R, Bucay N, Arakawa T (1997). Molecular characterization of ALK, a receptor tyrosine kinase expressed specifically in the nervous system. Oncogene.

[b3] Morris SW, Kirstein MN, Valentine MB, Dittmer KG, Shapiro DN, Saltman DL (1994). Fusion of a kinase gene, ALK, to a nucleolar protein gene, NPM, in non-Hodgkin’s lymphoma. Science.

[b4] Delsol G, Lamant L, Mariame B, Pulford K, Dastugue N, Brousset P (1997). A new subtype of large B-cell lymphoma expressing the ALK kinase and lacking the 2;5 translocation. Blood.

[b5] Griffin CA, Hawkins AL, Dvorak C, Henkle C, Ellingham T, Perlman EJ (1999). Recurrent involvement of 2p23 in inflammatory myofibroblastic tumors. Cancer Res.

[b6] Lamant L, Pulford K, Bischof D, Morris SW, Mason DY, Delsol G (2000). Expression of the ALK tyrosine kinase gene in neuroblastoma. Am. J. Pathol.

[b7] Murugan AK, Xing MZ (2011). Anaplastic thyroid cancers harbor novel oncogenic mutations of the ALK gene. Cancer Res.

[b8] van Gaal JC, Flucke UE, Roeffen MH, de Bont ES, Sleijfer S, Mavinkurve-Groothuis AM (2012). Anaplastic lymphoma kinase aberrations in rhabdomyosarcoma: clinical and prognostic implications. J. Clin. Oncol.

[b9] Soda M, Choi YL, Enomoto M, Takada S, Yamashita Y, Ishikawa S (2007). Identification of the transforming EML4-ALK fusion gene in non-small-cell lung cancer. Nature.

[b10] Dirks WG, Fahnrich S, Lis Y, Becker E, MacLeod RA, Drexler HG (2002). Expression and functional analysis of the anaplastic lymphoma kinase (ALK) gene in tumor cell lines. Int. J. Cancer.

[b11] Perez-Pinera P, Chang Y, Astudillo A, Mortimer J, Deuel TF (2007). Anaplastic lymphoma kinase is expressed in different subtypes of human breast cancer. Biochem. Biophys. Res. Commun.

[b12] Jazii FR, Najafi Z, Malekzadeh R, Conrads TP, Ziaee AA, Abnet C (2006). Identification of squamous cell carcinoma associated proteins by proteomics and loss of beta tropomyosin expression in esophageal cancer. World J. Gastroenterol.

[b13] Du XL, Hu H, Lin DC, Xia SH, Shen XM, Zhang Y (2007). Proteomic profiling of proteins dysregulted in Chinese esophageal squamous cell carcinoma. J. Mol. Med.

[b14] Wang WY, Gu L, Liu WP, Li GD, Liu HJ, Ma ZG (2011). ALK-positive extramedullary plasmacytoma with expression of the CLTC-ALK fusion transcript. Pathol. Res. Pract.

[b15] Debelenko LV, Raimondi SC, Daw N, Shivakumar BR, Huang D, Nelson M (2011). Renal cell carcinoma with novel VCL-ALK fusion: new representative of ALK-associated tumor spectrum. Mod. Pathol.

[b16] Lin E, Li L, Guan Y, Soriano R, Rivers CS, Mohan S (2009). Exon array profiling detects EML4-ALK fusion in breast, colorectal, and non-small cell lung cancers. Mol. Cancer Res.

[b17] Lipson D, Capelletti M, Yelensky R, Otto G, Parker A, Jarosz M (2012). Identification of new ALK and RET gene fusions from colorectal and lung cancer biopsies. Nat. Med.

[b18] Gandhi L, Janne PA (2012). Crizotinib for ALK-rearranged non-small cell lung cancer: a new targeted therapy for a new target. Clin. Cancer Res.

[b19] Choi YL, Soda M, Yamashita Y, Ueno T, Takashima J, Nakajima T (2010). EML4-ALK mutations in lung cancer that confer resistance to ALK inhibitors. N. Engl. J. Med.

[b20] Sasaki T, Koivunen J, Ogino A, Yanagita M, Nikiforow S, Zheng W (2011). A novel ALK secondary mutation and EGFR signaling cause resistance to ALK kinase inhibitors. Cancer Res.

[b21] Doebele RC, Pilling AB, Aisner DL, Kutateladze TG, Le AT, Weickhardt AJ (2012). Mechanisms of resistance to crizotinib in patients with ALK gene rearranged non-small cell lung cancer. Clin. Cancer Res.

[b22] Katayama R, Shaw AT, Khan TM, Mino-Kenudson M, Solomon BJ, Halmos B (2012). Mechanisms of acquired crizotinib resistance in ALK-rearranged lung Cancers. Sci. Transl. Med.

[b23] Gambacorti-Passerini C, Messa C, Pogliani EM (2011). Crizotinib in anaplastic large-cell lymphoma. N. Engl. J. Med.

[b24] Gambacorti-Passerini C, Farina F, Stasia A, Redaelli S, Ceccon M, Mologni L (2014). Crizotinib in advanced, chemoresistant anaplastic lymphoma kinase-positive lymphoma patients. J. Natl. Cancer Inst.

[b25] Mologni L (2012). Inhibitors of the anaplastic lymphoma kinase. Expert Opin. Investig. Drugs.

[b26] Redaelli S, Piazza R, Rostagno R, Magistroni V, Perini P, Marega M (2009). Activity of bosutinib, dasatinib, and nilotinib against 18 imatinib-resistant BCR/ABL mutants. J. Clin. Oncol.

[b27] Camidge D, Bazhenova L, Salgia R, Weiss G, Langer C, Shaw A (2013). First-in-human dose-finding study of the ALK/EGFR inhibitor AP26113 in patients with advanced malignancies: updated results. J. Clin. Oncol.

[b28] Kodama T, Tsukaguchi T, Yoshida M, Kondoh O, Sakamoto H (2014). Selective ALK inhibitor alectinib with potent antitumor activity in models of crizotinib resistance. Cancer Lett.

[b29] Friboulet L, Li N, Katayama R, Lee CC, Gainor JF, Crystal AS (2014). The ALK inhibitor ceritinib overcomes crizotinib resistance in non-small cell lung cancer. Cancer Discov.

[b30] Iwama E, Okamoto I, Harada T, Takayama K, Nakanishi Y (2014). Development of anaplastic lymphoma kinase (ALK) inhibitors and molecular diagnosis in ALK rearrangement-positive lung cancer. Onco Targets Ther.

[b31] Heuckmann JM, Balke-Want H, Malchers F, Peifer M, Sos ML, Koker M (2012). Differential protein stability and ALK inhibitor sensitivity of EML4-ALK fusion variants. Clin. Cancer Res.

[b32] Gorre ME, Mohammed M, Ellwood K, Hsu N, Paquette R, Rao PN (2001). Clinical resistance to STI-571 cancer therapy caused by BCR-ABL gene mutation or amplification. Science.

[b33] Tamborini E, Bonadiman L, Greco A, Albertini V, Negri T, Gronchi A (2004). A new mutation in the KIT ATP pocket causes acquired resistance to imatinib in a gastrointestinal stromal tumor patient. Gastroenterology.

[b34] Kobayashi S, Boggon TJ, Dayaram T, Janne PA, Kocher O, Meyerson M (2005). EGFR mutation and resistance of non-small-cell lung cancer to gefitinib. N. Engl. J. Med.

[b35] Pao W, Miller VA, Politi KA, Riely GJ, Somwar R, Zakowski MF (2005). Acquired resistance of lung adenocarcinomas to gefitinib or erlotinib is associated with a second mutation in the EGFR kinase domain. PLoS Med.

[b36] Ignatius Ou SH, Azada M, Hsiang DJ, Herman JM, Kain TS, Siwak-Tapp C (2014). Next-generation sequencing reveals a novel NSCLC ALK F1174V mutation and confirms ALK G1202R mutation confers high-level resistance to alectinib (CH5424802/RO5424802) in ALK-rearranged NSCLC patients who progressed on crizotinib. J. Thorac. Oncol.

[b37] Azam M, Latek RR, Daley GQ (2003). Mechanisms of autoinhibition and STI-571/imatinib resistance revealed by mutagenesis of BCR-ABL. Cell.

[b38] Awad MM, Katayama R, McTigue M, Liu W, Deng YL, Brooun A (2013). Acquired resistance to crizotinib from a mutation in CD74-ROS1. N. Engl. J. Med.

[b39] Sun H, Li Y, Li D, Winchester CL, Dalal K, Giacalone NJ (2013). Insight into crizotinib resistance mechanisms caused by three mutations in ALK tyrosine kinase using free energy calculation approaches. J. Chem. Inf. Model.

[b40] Sun HY, Ji FQ (2012). A molecular dynamics investigation on the crizotinib resistance mechanism of C1156Y mutation in ALK. Biochem. Biophys. Res. Commun.

[b41] Ceccon M, Mologni L, Giudici G, Piazza R, Pirola A, Fontana D (2014). Treatment efficacy and resistance mechanisms using the second-generation Alk inhibitor Ap26113 in human Npm-Alk-positive anaplastic large cell lymphoma. Mol. Cancer Res.

