# Supplementary figures and images for: Activity of second-generation ALK inhibitors against crizotinib-resistant mutants in an NPM-ALK model compared to EML4-ALK
Source: Cancer Med. 2015 Feb 26;4(7):953–65. doi: 10.1002/cam4.413 (PMC4529334; doi:10.1002/cam4.413)

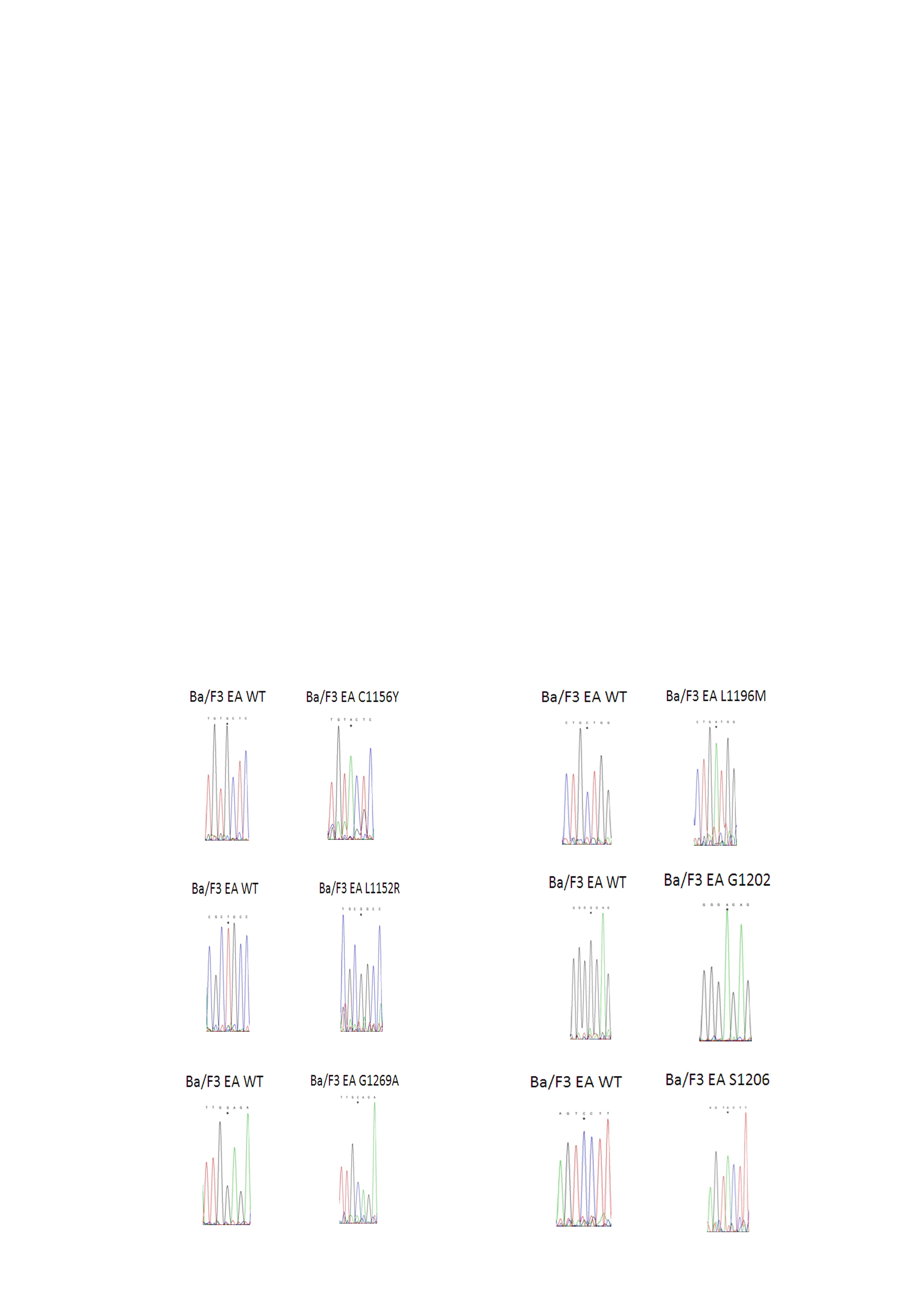

Supplement: Supplementary file 1 [file cam40004-0953-sd1.tif]

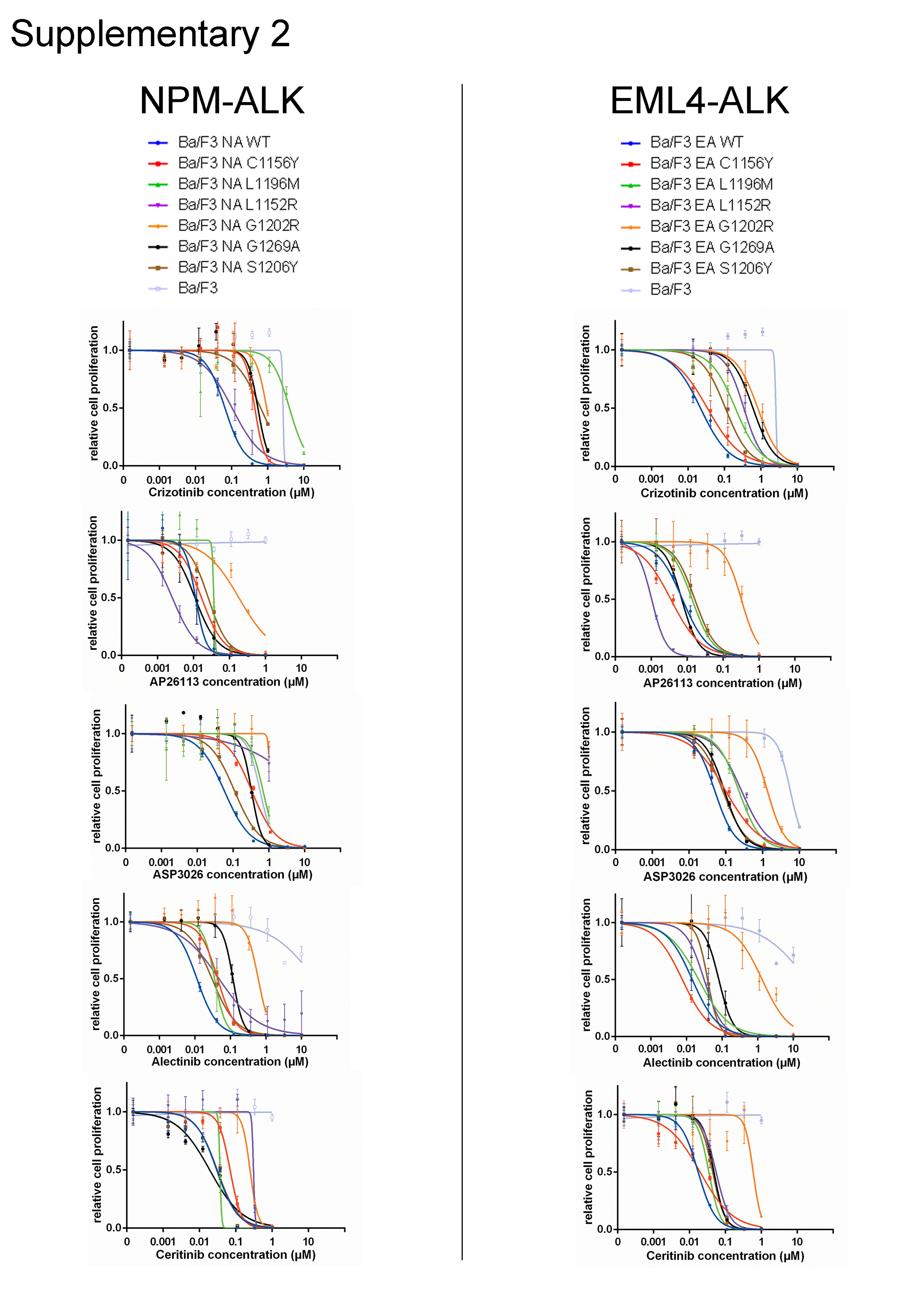

Supplement: Supplementary file 2 [file cam40004-0953-sd2.tif]
